# Effects of spermine NONOate and ATP on the thermal stability of hemoglobin

**DOI:** 10.1186/2046-1682-5-16

**Published:** 2012-08-28

**Authors:** Rasha Bassam, Juergen Hescheler, Ayseguel Temiz-Artmann, Gerhard M Artmann, Ilya Digel

**Affiliations:** 1 Institute of Bioengineering (IFB), Aachen University of Applied Sciences, 52428 Juelich, Germany; 2 Institute of Neurophysiology, University of Cologne, 50931 Cologne, Germany

## Abstract

**Background:**

Minor changes in protein structure induced by small organic and inorganic molecules can result in significant metabolic effects. The effects can be even more profound if the molecular players are chemically active and present in the cell in considerable amounts. The aim of our study was to investigate effects of a nitric oxide donor (spermine NONOate), ATP and sodium/potassium environment on the dynamics of thermal unfolding of human hemoglobin (Hb). The effect of these molecules was examined by means of circular dichroism spectrometry (CD) in the temperature range between 25°C and 70°C. The alpha-helical content of buffered hemoglobin samples (0.1 mg/ml) was estimated via ellipticity change measurements at a heating rate of 1°C/min.

**Results:**

Major results were: 1) spermine NONOate persistently decreased the hemoglobin unfolding temperature _*Tu*_irrespectively of the ^Na + ^/^K + ^environment, 2) ATP instead increased the unfolding temperature by 3°C in both sodium-based and potassium-based buffers and 3) mutual effects of ATP and NO were strongly influenced by particular buffer ionic compositions. Moreover, the presence of potassium facilitated a partial unfolding of alpha-helical structures even at room temperature.

**Conclusion:**

The obtained data might shed more light on molecular mechanisms and biophysics involved in the regulation of protein activity by small solutes in the cell.

## Background

One intrinsic property of a protein is its ability to adopt and maintain a certain range of characteristic shapes within a given scope of temperature, pH, pressure, etc. Mostly, these structural features are vitally important for the protein’s functioning. Therefore, ability of some low-molecular weight compounds to shift a protein’s conformational equilibrium by binding proteins is used by cells to orchestrate virtually all aspects of their life and even death. Today, numerous cellular signaling pathways are known, involving small molecules as key mediators, regulators and messengers but the very intimate structural aspects of signaling interactions remain mostly undiscovered. Some very interesting studies were recently reported by [1] but the progress in this direction is rather slow because of technical and experimental difficulties (the events of interest occur at single-molecular level and have very short durations) and also because of certain disregard from cellular biologists.

A few years ago we reported for the first time a temperature-driven structural transition in mammalian hemoglobins (Hb) that was closely related to the organism’s body temperature [2-6]. Those data were collected using various techniques: micropipette aspiration [7], CD spectroscopy [2], quasi-elastic light scattering [4] as well as NMR spectroscopy and colloid-osmotic pressure measurements [3]. The phenomenon of hemoglobin’s structure transition was later confirmed by partnering research groups using neutron scattering [8,9]. We also addressed potential modulating roles of the chemical environment, especially pH and ^Ca2 + ^concentration, in the manifestation of the discovered effect [5].

Nitric oxide (NO) has long been known to play important roles in physiology, pathology and pharmacology, being involved in numerous biological processes [10]. Evidence is growing concerning multiple chemical mechanisms of nitric oxide interaction with proteins. They mostly involve the cysteine residues in proteins. A process of introduction of the nitric group into a protein molecule, known as nitrosylation, appears to be a widely used mechanism of cellular metabolic regulation but its exact implication in protein dynamics is still unclear [11].

Another ubiquitous cellular messenger, adenosine 5’-triphosphate (ATP) exerts most of its actions by interacting with specialized proteins, both inside and outside the cell [12]. Although characteristic domains like the Rossmann fold are believed to be necessary for binding ATP molecules [13], some studies indicate that ATP molecule affects also many proteins, lacking this “standard” ATP-binding sites. For example, the addition of ATP to a diluted solution of collagen caused an appearance of segment-long-spacing aggregates [14]. Also, the change in the concentration of ATP in RBCs resulted in alterations in Hb oxygen affinity [15]. In red blood cells, the concentration of ATP is rather high (0.2-2.0 mM) and therefore might influence properties of hemoglobin considerably [16].

Regarding the ionic environment, ^Na + ^(native ionic radius 0.95 *Å*) and ^K + ^(1.33 *Å*) demonstrate complex hydration behavior, influencing the hydrogen bond network of water in distinctly different ways. It was found that water molecules in the hydration shell of ^K + ^ are orientationally more disordered than those hydrating a ^Na + ^ion and are inclined to orient their dipole moments tangentially to the hydration sphere [17,18]. In proteins, charged and polar groups interact with these ions in quite different ways too. Potassium generally exhibits stronger affinity to proteins as compared to sodium [17,19]. Protein’s preferential affinity to potassium ions can possibly contribute to their accumulation inside the living cell [12,20].

According to our hypothesis and as further logical development of those studies we recently focused our attention on the effects of regulators such as nitric oxide, ATP and others on the conformational and hydrodynamic properties of proteins in various ionic environments. Using the quasi-elastic light scattering technique, we found that nitric oxide in micromolar concentrations decreased the aggregation temperature of hemoglobin significantly. A comparable concentration of ATP, instead, counteracted thermal denaturation. The magnitude and direction of the observed effects strongly depended on concentrations of ^K + ^ and ^Na + ^ in the solution.

Here we present data on the effects of the NO-donor spermine NONOate, ATP, as well as sodium and potassium ions on the thermal unfolding of human hemoglobin. The study was based on temperature scans of hemoglobin solutions with circular dichroism (CD) spectroscopy. The CD technique has established itself as a standard tool is studying polymers because of its sensitivity to conformational changes [2,21].

## Methods

### Buffers

Two different buffers were used for sample preparation in order to examine the role of ^K + ^/^Na + ^balance in the medium: sodium-based phosphate buffered saline (137 mM NaCl, 2.7 mM KCl, 8.1 mM Na_2_HPO_4_, 1.76 mM NaH_2_PO_4_), further referred to as Na-buffer, and its potassium-based counterpart (termed bellow as K-buffer) composed of 0.1 M KCl, 61.3 mM K_2_HPO_4_ and 5.33 mM KH_2_PO_4_. Both buffers had pH 7.4 and osmolarity 290 ±10 mosm/l.

### Sample preparation

Human hemoglobin was prepared from fresh erythrocytes (RBCs) obtained from healthy volunteers [22]. Briefly, 50 *μ*L of heparinized blood were collected from each donor’ fingertip. Obtained RBCs were washed twice in 1 mL of Na-buffer or K-buffer solution, respectively, using centrifugation at 1000 g for 5 minute at 25°C. The RBC pellet was hemolysed by adding 200 *μ*L of distilled water and 5 min vigorous shaking. Afterwards, the samples were centrifuged at 18000 g for 5 minute and 25°C to remove RBC ghosts. Finally, ionic strength and pH of the obtained hemoglobin solutions were adjusted to mimic physiological values mosm/l 10 ± 290 and pH 7.4 [22]. The samples were stored at 4°C until the measurement started (typically 2 to 4 hours).

Prior to the CD measurement, the samples were twice syringe-filtered through a 0.2 *μ*m WhatmanⓇ nitrocellulose filters to remove large particles. The concentration of Hb was adjusted to 0.1 mg/mL and controlled photometrically as described elsewhere [15,23] using a UV-Vis V-550 JascoⓇ spectrophotometer (Jasco Labor- und Datentechnik GmbH, Gross-Umstadt, Germany).

### Sample treatment with spermine NONOate and ATP

The use of nitric oxide donors allows avoiding many difficulties inherent in gaseous nitric oxide (II) applications [24]. Nucleophilic complexes of NO with amines (NONOates) appear to meet most of research criteria [25]. These compounds are self-decomposing in solution producing 2 mole of NO per mole of the substrate. In this study spermine NONOate was chosen for simplicity in handling, storage stability and its convenient half-life in solution (about 39 min at 37°C) [10,24].

Just before the measurement, freshly prepared spermine NONOate stock solution in a corresponding buffer was injected through a 0.2 *μ*m filter into the scintillation vial containing the sample to achieve the Hb/NONOate molecular ratio close to 1:1 during the initial phase of NONOate’s decay). In the control group, the same volume of the corresponding pure buffer was added.

In a similar manner, freshly prepared ATP stock solution was introduced into the Hb sample to achieve the Hb/ATP molecular ratio close to 1:1 immediately before the measurement. Spermine NONOate (N-(2-Aminoethyl)-N-(2-hydroxy-2-nitrosohydrazino)-1,2-ethylene-diamine) of 98% purity was purchased from MerckⓇ KGaA, Darmstadt, Germany. Adenosine-5-triphosphate (di-sodium salt) of 98% purity was purchased from Carl Roth GmbH, Karlsruhe, Germany.

### Circular dichroism measurement

The far-UV CD spectra were measured at the J-815 Circular Dichroism Spectrometer (Jasco Labor- und Datentechnik GmbH, Gross-Umstadt, Germany). Temperature was adjusted through a PC-controlled built-in Peltier element. Thermal unfolding of hemoglobin was followed between 25°C and 70°C. The samples were initially equilibrated at 25°C for 10 minute and then the temperature has been increased gradually up to 70°C with a rate 1°C/min. During this, wavelengths scans (wavelength steps 1 nm, average time = 4 s, time response = 2 s, band width = 1 nm) were performed in the UV-region between 190 and 260 nm. Blank spectra of buffer solutions were subtracted from the Hb spectra at each temperature point and the offset was corrected at 250 nm. At 222 nm, CD spectra are most sensitive to changes in the alpha-helical content of proteins [26]. Therefore, absolute ellipticities at 222 nm were taken as a measure of the alpha-helical content of the protein [27] and plotted against temperature. Decrease of ellipticity magnitude at 222 nm was interpreted as loss of alpha-helical structures (unfolding).

Generally, the CD-measurements can be complicated by the fact that typical aqueous buffer systems (phosphate, sulfate, carbonate, and acetate) strongly absorb in the UV-range where sample’s structural features exhibit differential absorption of circularly polarized light. These groups usually interfere with the CD-signal at concentrations exceeding 100 mM. Therefore, concentration of phosphates in our samples was always kept bellow mM 100.

### Experimental data analysis

For statistical analysis, each experimental group was measured at least as triplicate. The raw CD data (ellipticity as function of temperature) were initially extracted as ASCII text and processed by the MATLABⓇ software (Math Works, Massachusetts, USA). Further analysis and visualization of the data were done using OriginPro 8Ⓡ (OriginLab Corporation, USA). To compare the groups, mean values and corresponding standard deviations were calculated.

## Results

**Figure 1:**
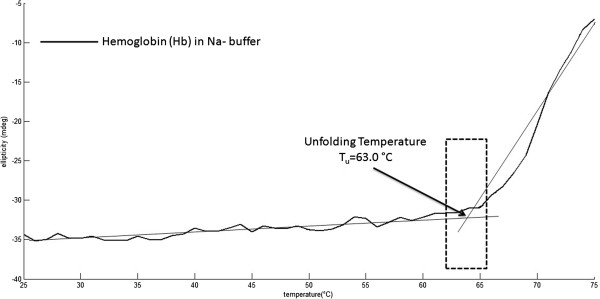
**Best-fit tangential line method for protein unfolding temperature calculation.** Calculation of the unfolding temperature (*T*_*u*_) using the best-fit tangential lines method, which is the intersection point between the first gentle line slope and the second steep slope.

The measured far-UV CD spectra of Hb samples expectedly displayed a typical alpha-helical signature with local minima at 208 and 222 nm [28]. Upon raising the temperature from 25°C to 70°C by equidistant temperature steps, the ellipticity response was far from linear (Figure 1, a-d). When the ellipticities at 222 nm were plotted versus temperature, two distinct kink points usually appeared so that the curves had a characteristic s-shape (with increasing temperature: ^1*st*^ gentle slope, steep slope, ^2*nd*^ gentle slope). The unfolding temperature, _*Tu*_, was calculated as the intersection point between the best-fit tangential lines (Figure 1) drawn to the 1st gentle slope and the steep slope parts of the curve [4].

### Effects of spermine NONOate and ATP on _*Tu*_of Hb samples in Na-buffer

**Figure 2:**
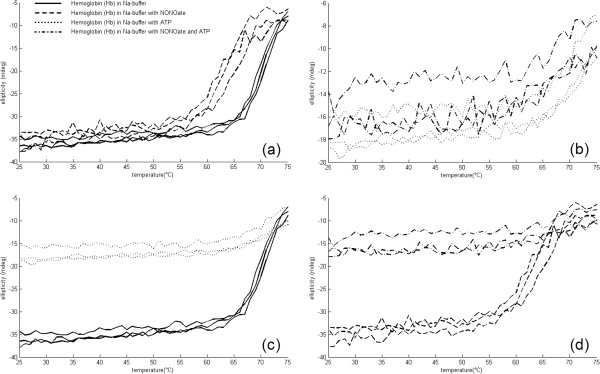
**Influence of ATP and spermine NONOate on unfolding of Hb samples prepared in Na-buffer.** Influence of ATP and spermine NONOate on unfolding of Hb samples prepared in Na-buffer. **a)** control groups vs. NONOate groups; **b)** ATP groups vs. ATP+NONOate groups; **c)** control groups vs. ATP groups; **d)** NONOate groups vs. ATP+NONOate groups.

For the control samples prepared in Na-buffer, _*Tu*_was found to be 64.0 ± 0.6°C, slightly varying from individual to individual. Addition of spermine NONOate significantly decreased the unfolding temperature: _*Tu*_ was found to be 56.0 ± 1.3°C (Figure 2a). Upon addition of ATP the _*Tu*_ on the contrary, was shifted towards 66.0 ± 1.2°C (Figure 2c). Remarkably, when ATP and nitric oxide (NO) appeared in the solution simultaneously, _*Tu*_ values did not differ from those obtained for ATP alone (65.5 ± 1.3°C) (Figure 2b, d). Table 1 shows the effect of NONOate and ATP in Na-buffer on Hb unfolding.

**Table 1:** Effect of NONOate and ATP in Na-buffer on Hb unfolding

**Sample**	**unfolding temperature**	**Ellipticity**	**SD**
	**(**_***Tu***_**) (°C)**	**(mean)**	
Control-Na Buffer	64.0	-31.7	0.6
NONOate	56.0	-31.4	1.3
ATP	66.0	-15.1	1.2
NONOate+ATP	65.0	-13.1	1.3

### Effects of spermine NONOate and ATP on _*Tu*_of Hb samples in K-buffer

**Figure 3:**
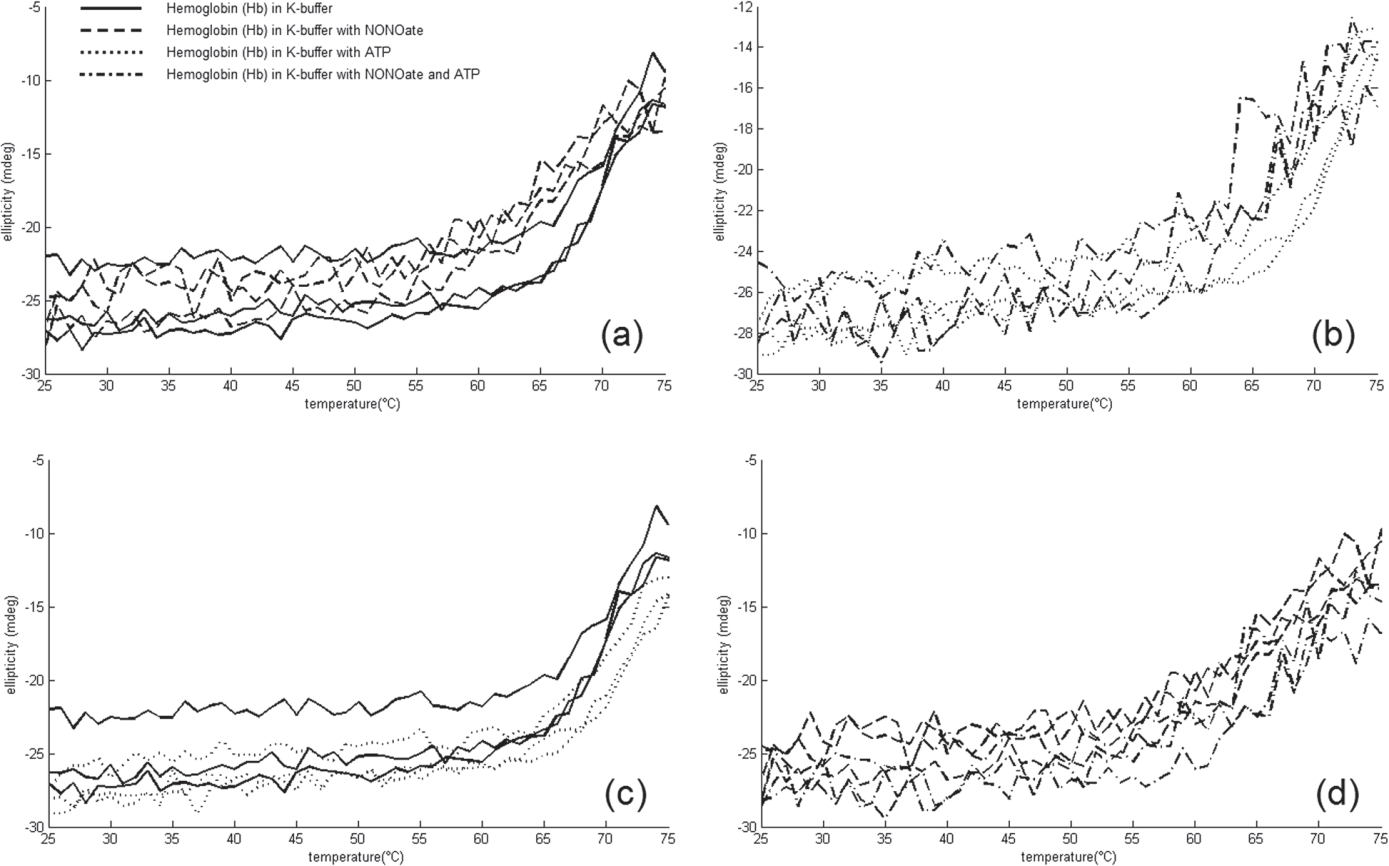
**Influence of ATP and spermine NONOate on unfolding of Hb samples prepared in K-buffer.** Influence of ATP and spermine NONOate on unfolding of Hb samples prepared in K-buffer. **a)** control groups vs. NONOate groups; **b)** ATP groups vs. ATP+NONOate groups; **c)** control groups vs. ATP groups; **d)** NONOate groups vs. ATP+NONOate groups.

In case of using the potassium-based buffer instead of the sodium-based one, unfolding of the samples occurred at 62.0 ± 1.1°C, whereas the addition of the NONOate resulted in a shift of _*Tu*_towards 59.0 ± 1.7°C (Figure 3a). Adding ATP to the sample increased its thermal stability up to 65.0 ± 1.4°C (Figure 3c). When ATP and nitric oxide donor were applied together, the _*Tu*_values did not significantly differ from those of the NONOate-group 61.0 ± 1.1°C, (Figure 3b, d). Like the Na-samples, the potassium-based solutions also demonstrated the s-shaped profile of thermal unfolding of human hemoglobin. Table 2 shows the effect of NONOate and ATP in K-buffer on Hb unfolding.

**Table 2:** Effect of NONOate and ATP in K-buffer on Hb unfolding

**Sample**	**Unfolding temperature**	**Ellipticity**	**SD**
	**(**_***Tu***_**) (°C)**	**(mean)**	
Control- K Buffer	62.0	-22.6	1.1
NONOate	59.0	-21.5	1.7
ATP	65.0	-23.8	1.4
NONOate	61.0	-23.9	1.1

### Effect of sample composition on Hb’s ellipticity measured at 25°C

Scrutiny at initial parts of the curves on Figures 2 and 3 suggests that not only thermal unfolding but also Hb’s initial alpha-helical content was significantly impacted by the molecules present in the solution. Initial sample ellipticities measured at 222 nm for K-buffer were all lying approximately between -22.5 mdeg and -27.5 mdeg irrespectively to ATP and NONOate content. The ATP-free Na-based samples had typical ellipticities in the range between -33 mdeg and -37 mdeg, whereas the addition of ATP always caused the ellipticity raise (i.e. less negative) to -14 mdeg to -18 mdeg, indicating partial unfolding of alpha-helices.

## Discussion

Without any doubt, protein conformational properties depend on multiple physico-chemical factors, such as temperature, pH, ionic strength as well as presence of numerous protein-binding groups and molecules [29]. Protein unfolding with subsequent aggregation plays a crucial role in biology and in many applications of protein science and medical engineering [30]. Regulation of protein stability in the cell using small organic and inorganic molecules has been evolutionary proved as a rapid, reversible, and tunable method of metabolic control. Despite its biological importance, little is known about the mechanisms and potential signaling pathways involved in the formation of molecular aggregates [31]. Among numerous low-molecular mediators of cellular activity, nitric oxide and ATP have attracted our interest, because of their ubiquity in living systems. Our attempts to study hemoglobin temperature denaturation were related to our earlier interesting findings on unfolding “abnormalities” around body temperature [2-6].

The use of CD spectroscopy is generally of great advantage in protein structural studies [32,33]. CD is intrinsically very sensitive to changes in the secondary structure of proteins [31,34]. It is to a lower extent affected by molecular aggregation and protein concentration effects [35]. Therefore we attribute the observed ellipticity changes predominantly to the partial thermal unfolding of hemoglobin *α*-helices, and, in much lesser extent, to changes in aggregation and/or in molecular size and shape.

We compared the data reported here with our data obtained previously with dynamic light scattering (DLS) using identical hemoglobin samples. The aggregation temperature _*Ta*_ obtained with DLS and the unfolding temperature _*Tu*_from CD measurements, respectively, were very close to each other [2,7].

One possible mechanism of the observed “destabilizing” effect of NO on hemoglobin might be its penetration into the protein’s hydrophobic core [36]. Such “loosening” of the Hb structure caused by nitric oxide could contribute to accelerating its unfolding. Furthermore we may speculate about the action of nitric oxide on the hydration shell of hemoglobin. Such action would become plausible considering a re-arrangement of the hydrogen bond network of the vicinal water [3]. Revealing of particular mechanisms must remain subject of our future studies.

The addition of ATP in our experiments systematically resulted in an increase of the Hb’s unfolding temperature _*Tu*_ by approximately 2°C. We interpreted the effects of ATP in both sodium- and potassium-based buffers as “moderate secondary structure stabilization”. Moreover, if ATP and NONOate were added simultaneously to the K-based Hb samples, no “destabilization” effect of NONOate was observed, but rather a slight increase of the Hb’s unfolding temperature. In contrary to that, in the Na-based Hb samples simultaneous occurrence of NONOate and ATP resulted in dramatic Hb unfolding. These data strongly suggest involvement of Na/K-environment in Hb stabilization/destabilization previously hypothesized[18].

Physiologically, that would mean, for example, that due to big differences in Na/K composition between extracellular and intracellular media a protein might have very different levels of unfolding (i.e. conformational shape being inside or outside the cell. This, in turn might result in exposure/hiding of certain recognition sites, leading to totally different signaling outcomes, for example, it could contribute to protein sequestering mechanisms in the organism.

It has been previously argued by several independent groups that sodium as a “structure stabilizer” and potassium as a “structure disrupter” have different affinity to proteins, especially to denatured proteins [18,19,37]. Moreover, many residues show a preference for ^ + ^K -binding as compared to Na ^+ ^[37]. Our results support these observations by bringing further evidence that potassium has stronger impact on hemoglobin unfolding and destabilization. In other words we must assume that inside red blood cells where the potassium concentration is very high the hemoglobin structure is quite loosened which could help explain the low cytosolic viscosity and consequently the fast (deformation) of red blood cells at changing shear forces observed in vivo.

## Conclusion

We examined the unfolding of hemoglobin when implementing NO-donor spermine NONOate, ATP, and the combination of these compounds with elevating temperature in different buffers. Our main observations were: 1) the nitric oxide donor systematically caused a reduction of hemoglobin’s unfolding temperature, _*Tu*_; 2) cationic composition of the medium affected the manifestation of the ATP- and NO- effects, and 3) the effect of ATP on the _*Tu*_ of Hb shows distinctly different outcomes: In pure Na-buffer as well as in pure K-buffer ATP stabilized the protein’s secondary structure and shifted _*Tu*_by approximately 2°C toward higher temperatures. Simultaneous addition of NONOate and ATP led to an apparent compensation each other’s structural effects. ATP in sodium-based medium facilitated the unfolding of Hb.

We attribute the unfolding of Hb in the presence of NONOate to the in-situ generated nitric oxide since the magnitude of the effects strongly correlated with the kinetics of the NONOate decay in aqueous solutions. Hereby we claim a new biophysical aspect of nitric oxide action, defined as facilitation of thermal unfolding and thus destabilization of the secondary structure of hemoglobin.

## Competing interests

The authors have declared that no competing interest exists.

## Author’s contributions

RB writes the whole paper and performed the experimental and analytical work. ID and GMA review and proof-editing the paper and supervising the whole research work. TAA and JH review and comment on the paper. All authors read and approved the final manuscript.
